# Treatment pathways of lipid-lowering therapies in Germany 2016–2022

**DOI:** 10.1007/s00392-025-02686-5

**Published:** 2025-05-28

**Authors:** Julius L. Katzmann, Claudia Grellmann, Beate Leppert, Irina Müller-Kozarez, Martin Schulz, Ulrich Laufs

**Affiliations:** 1 Klinik und Poliklinik für Kardiologie, Universitätsklinikum Leipzig, Liebigstraße 20, 04103 Leipzig, Germany; 2 inav – privates Institut für angewandte Versorgungsforschung GmbH, Berlin, Germany; 3 Gesundheitsforen Leipzig GmbH, Leipzig, Germany; 4 Deutsches Arzneiprüfungsinstitut e. V. (DAPI), Berlin, Germany; 5 Institut für Pharmazie, Freie Universität Berlin, Berlin, Germany

**Keywords:** Statin, Ezetimibe, Bempedoic acid, PCSK9 inhibitor, Therapeutic inertia

## Abstract

**Background:**

Despite the availability of effective LDL cholesterol (LDL-C)-lowering drugs, only a minority of patients achieves the guideline-recommended treatment targets. This analysis describes treatment pathways of lipid-lowering therapy (LLT) in Germany.

**Methods:**

Health claims data were used to identify patients at high or very-high cardiovascular risk who received a LLT prescription 2016–2022. Treatment pathways and the time to switch or discontinue LLT were analysed for statins, ezetimibe, bempedoic acid (BA), and PCSK9 inhibitors (PCSK9i).

**Results:**

Out of 3,487,827 insured persons, 247,529 met the inclusion criteria. The most frequent first-line LLT were statins in 96.3%. Ezetimibe, BA, and PCSK9i were first-line LLT in only 0.9%, 0.061%, and 0.046%, respectively. Only few patients experienced a change in their treatment regimen following LLT initiation. Prescriptions of BA and PCSK9i were mainly second-, third-, or fourth-line add-on treatment. Termination of treatment with BA and PCSK9i was less frequent compared to statins and ezetimibe. The median time to treatment discontinuation was 1.45, 1.04, 0.60, and 2.45 years for statins, ezetimibe, BA, and PCSK9i, respectively, and the median time to switch therapy was 4.81 and 4.87 years for ezetimibe and PCSK9i, respectively (median not reached for statins and BA).

**Conclusions:**

Changes in LLT were only observed in a minority of patients. BA and PCSK9i were switched more frequently than statins and ezetimibe. BA was discontinued earlier, and PCSK9i later than the other agents. Continued efforts to maintain long-term adherence and overcome therapeutic inertia are needed to realise the potential of available LLT with proven cardiovascular benefit.

**Graphical abstract:**

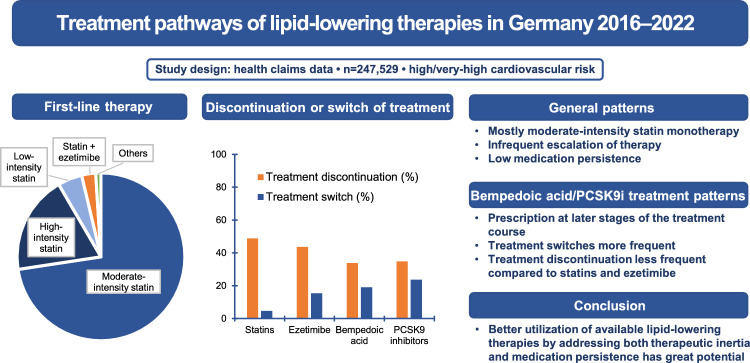

**Supplementary Information:**

The online version contains supplementary material available at 10.1007/s00392-025-02686-5.

## Introduction

The ESC/EAS guidelines for the management of dyslipidemias recommend individualized cardiovascular risk assessment and define treatment targets for low-density lipoprotein cholesterol (LDL-C) [1]. To achieve the LDL-C targets, stepwise treatment with statins, ezetimibe, bempedoic acid (BA), and PCSK9 inhibitors (PCSK9i) is recommended [1–3]. Randomized trials, registries, and simulation studies show that using the available treatment options to lower LDL-C, at least 90% of patients can achieve their LDL-C target [4–8]. However, it has been repeatedly observed that in clinical practice, only 20 to 30% of patients with elevated cardiovascular risk achieve the LDL-C target [9–11].

Low medication adherence and persistence, and therapeutic inertia partly explain this discrepancy. After treatment initiation, medication persistence gradually declines [12–15]. Furthermore, previous studies demonstrated that only a minority of patients experiences intensification of lipid-lowering therapy (LLT) despite uncontrolled LDL-C [16–18].

Combination LLT is frequently required to sufficiently lower LDL-C. Therefore, treatment intensification and long-term persistence to combination LLT are the key prerequisites to increase the proportion of patients attaining the LDL-C targets. Besides statins and ezetimibe, BA and PCSK9i are the latest additions to the lipid-lowering armamentarium and enable additional LDL-C lowering. The implementation of these drugs in daily practice is incompletely understood but may reveal opportunities for improvement of care.

Using health claims data of 4.1 million insured persons, the current analysis aimed to describe treatment pathways of LLTs in Germany and to compare the time to switch or termination of LLT for statins, ezetimibe, BA, and PCSK9i.

## Methods

### Data source

This analysis was based on health claims data from a German statutory health insurance database (“Deutsche Analysedatenbank für Evaluation und Versorgungsforschung” [DADB], administered by “Gesundheitsforen Leipzig GmbH”) which contains comprehensive claims data routinely collected by German statutory health insurance funds, beginning in 2013. Data from 15 statutory health insurers are included, representing about 4.1 million insured persons, corresponding to approximately 5% of all statutory health-insured persons in Germany (approximately 90% of the German population have statutory health insurance). The database contains anonymized information, but allows for longitudinal tracking of individuals.

The population in the database is fairly representative of the total statutory health-insured population, ensured by annual comparisons with data published by the German Federal Office for Social Insurance (“Bundesamt für Soziale Sicherung”, BAS). The DADB population is slightly younger than the total German statutory health-insured population, with the largest deviation for females aged 60 or older, but without differences in morbidity, since morbidity groups in the in- and outpatient setting are comparable to the data published by the BAS [19].

Ethical approval was not required, as all data were anonymized.

### Patient selection and study design

**Fig. 1:**
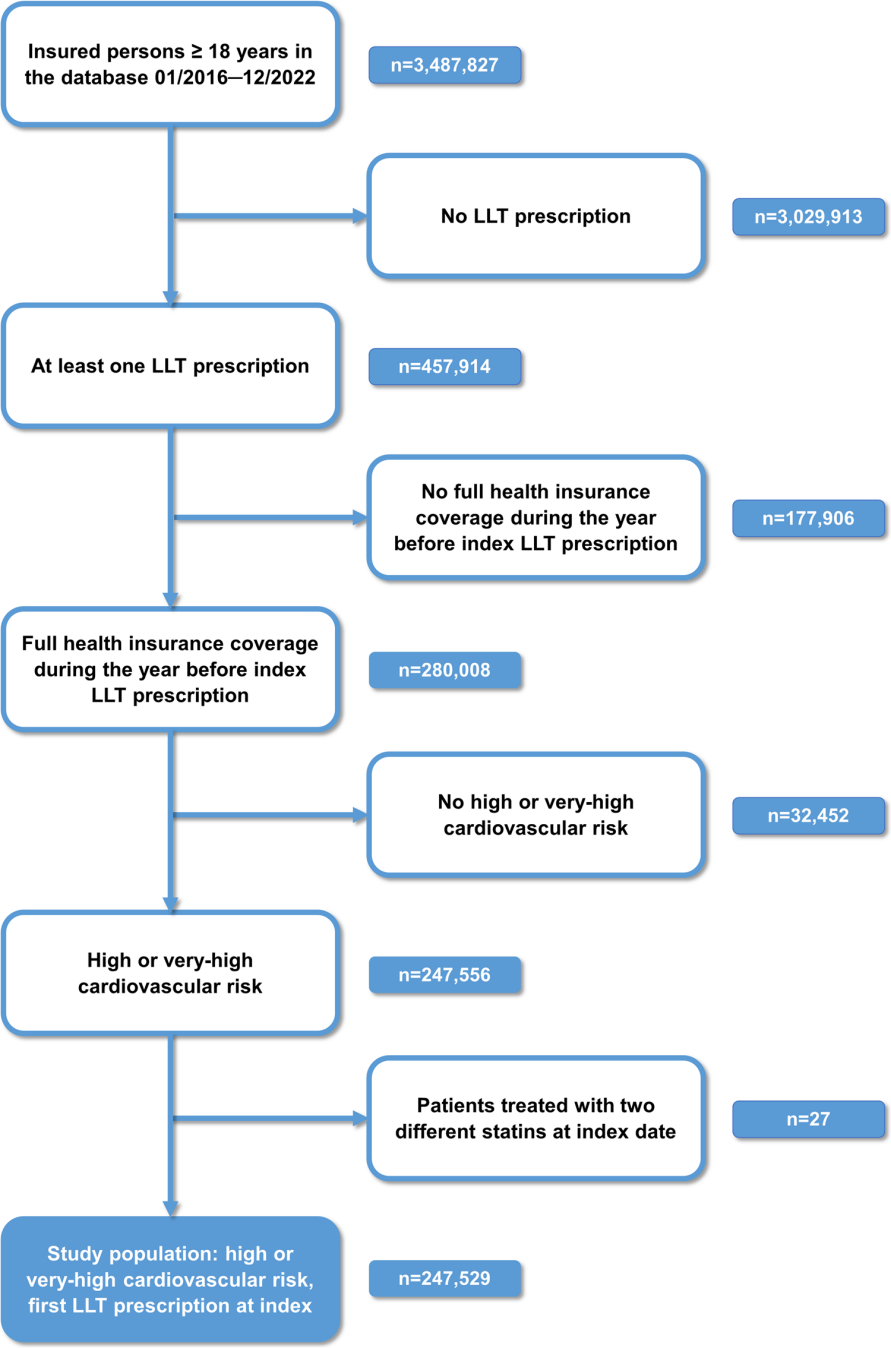
Flow chart of patient selection

For this non-interventional, longitudinal study, insured persons ≥ 18 years of age with available data in the period from 1 st January 2016 through 31 st December 2022 were included if they received a first prescription of LLT (based on an LLT-free period of one year before the index date), had full insurance coverage during the year before index LLT prescription, and had high or very-high cardiovascular risk. Patients treated with two different statins at index date were excluded (Fig. 1).

LLTs were identified according to the ATC codes, including fixed-dose combinations (Supplementary Table S1). The term “PCSK9 inhibitors” encompasses the monoclonal antibodies evolocumab and alirocumab, and the small-interfering RNA inclisiran. Statin intensity was defined according to the study by Fox et al. [18] (Supplementary Table S2). Cardiovascular risk was defined according to the ESC/EAS guidelines for the management of dyslipidemias 2019 [1]. The diagnosis and procedure codes used to define cardiovascular risk are detailed in Supplementary Table S3.

The treatment pathways of LLT are presented for all lipid-lowering agents and for treatment lines involving BA and PCSK9i separately as Sankey diagrams. The first line of treatment is the LLT medication prescribed at index. New treatment lines are indicated by a change or addition of a substance or change in intensity of statins. To maintain clarity, the diagrams are restricted to four lines of treatment, and rare treatment lines are summarized as indicated in the figure descriptions. The Sankey diagrams show prescription events, not individual patients. In patients with treatment gaps of one year or more after runout of a claim, defined on basis of the defined daily dose, plus potential hospitalizations in the observation period, a second record is created and patients are reassigned to first-line treatment.

The time to switch or termination of LLT is analysed with the Kaplan–Meier method separately for first-line treatment with statins, ezetimibe, BA, and PCSK9i. A switch of LLT is defined as addition of a new substance or replacement of the current substance by another. The time to switch therapy denotes the time between start of first- and second-line LLT. Treatment switches are required to occur within the grace period of 90 days after runout of a claim plus potential hospitalizations in the observation period, otherwise treatment is considered terminated. The term “treatment termination” therefore involves (1) treatment continuation after treatment breaks of more than 90 days, (2) treatment switches after treatment breaks of more than 90 days, and (3) “true” treatment termination.

For both switch and termination of treatment, patients are considered right censored if the observation period in the database ends while patients are on treatment (plus grace period of 90 days plus potential hospitalizations in the observation period).

All analyses were performed using the software Microsoft SQL Server Management Studio or the statistical software R.

## Results

### Baseline characteristics

Between 2016 and 2022, n = 3,487,827 insured persons ≥ 18 years of age were identified. Of those, n = 457,914 received at least one prescription of LLT. After exclusion of patients without full health insurance coverage during the one year before the index LLT prescription, of patients not meeting the criteria for high or very-high cardiovascular risk, and of those receiving two different types of statins, n = 247,529 patients with n = 280,100 prescription events were included in the analyses. The flow-chart of patient selection is depicted in Fig. 1.

The included patients had a mean age (standard deviation [SD]) of 64.8 (11.8) years, 42.5% were female. Atherosclerotic cardiovascular disease was diagnosed in 59.7% in the calendar year of the index LLT prescription, mostly coronary artery disease (36.1% of all patients). The most prevalent cardiovascular risk factors were hypertension in 87.0% of patients and diabetes mellitus (34.1%). Almost all patients were classified as very-high cardiovascular risk (97.1%; Table 1).

**Table 1:** Baseline characteristics

General	
N	247,529
Prescription events (n)	280,100
Female (%)	42.5
Age (mean [SD] in years)	64.8 (11.8)
Atherosclerotic cardiovascular disease (%)	59.7
Coronary artery disease	36.1
Percutaneous coronary intervention or coronary artery bypass graft	10.6
Cerebrovascular disease	16.8
Peripheral artery disease	22.8
Cardiovascular risk factors (%)	
Hypertension	87.0
Diabetes	34.1
Diabetes with target organ damage	15.5
Chronic kidney disease stage 3	7.6
Chronic kidney disease stage 4	2.2
Cardiovascular risk “high”^a^	1.3
Cardiovascular risk “very-high”^a^	97.1
First-line lipid-lowering therapy (%)	
Statin	
Moderate intensity	72.6
High intensity	19.0
Low intensity	4.7
Statin (any intensity) + ezetimibe	2.7
Ezetimibe mono	0.9
Bempedoic acid	0.061
PCSK9 inhibitor	0.046

^a^According to the 2019 ESC/EAS guidelines for the management of dyslipidaemias [1]. A small fraction of patients is neither classified as high or very-high cardiovascular risk, as risk estimation for patient selection was based on diagnoses before or together with the index lipid-lowering therapy prescription, whereas risk status analysis was based on the calendar year of the index prescription

### Treatment pathways of lipid-lowering therapy

The most frequently prescribed first-line LLTs were statins in 96.3% of cases, mostly at moderate intensity (Table 1). Combinations of statins and ezetimibe (both as fixed-dose combinations and as separate pills) represented 2.7% of first prescriptions, whereas ezetimibe as monotherapy was prescribed to 0.9% of patients, and BA and PCSK9i to 0.061 and 0.046%, respectively.

**Fig. 2:**
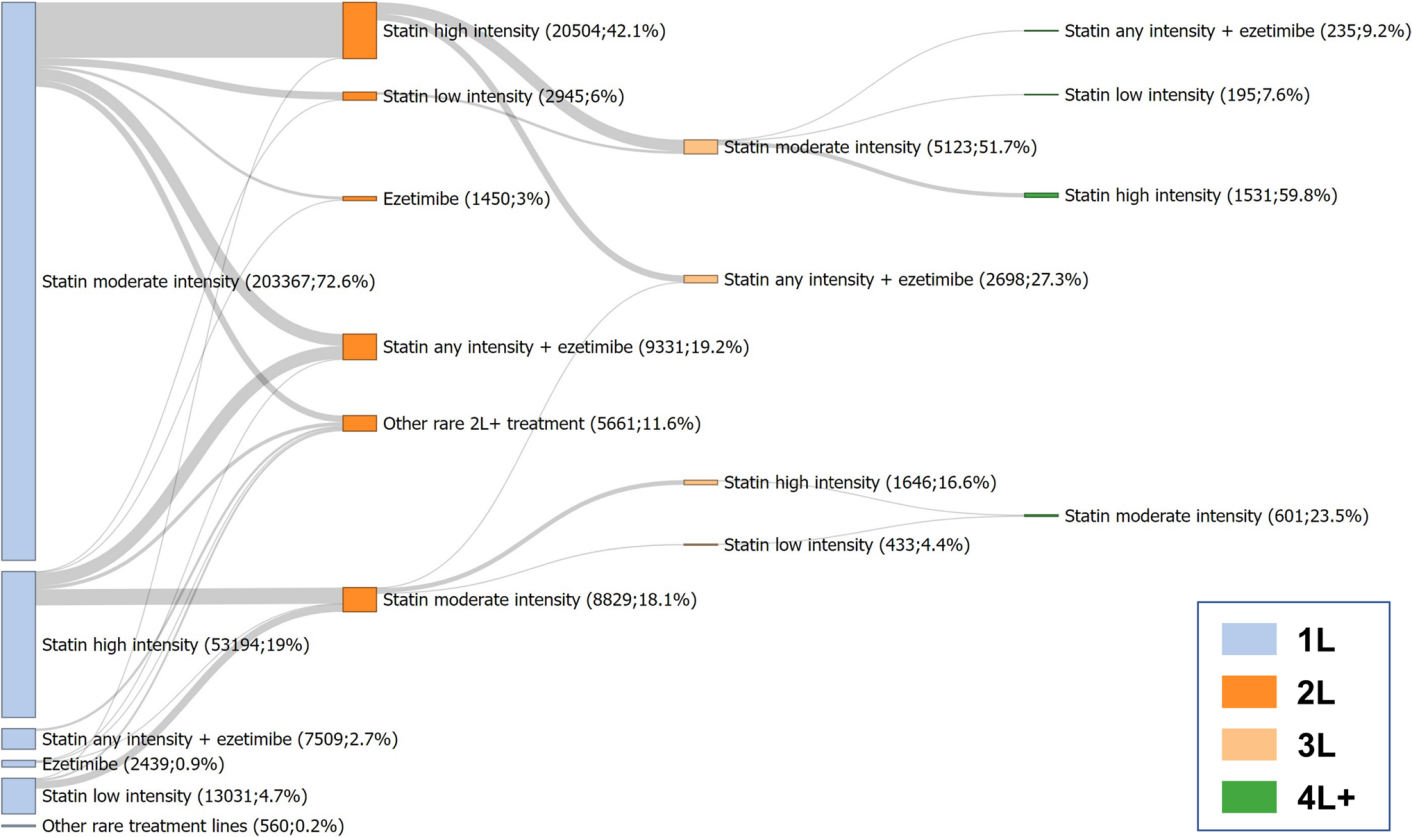
Treatment pathways of all lipid-lowering therapies. N = 280,100 events of lipid-lowering therapy initiation were analysed, with double counting of patients with treatment gaps of at least one year. Rare index treatments affecting less than 1% of patients are summarized as “other rare treatment lines”, rare 2L + treatments affecting less than 0.5% of patients are summarized as “other rare 2L + treatment”. *1L, 2L, …* first line, second line, …

The pathways for the different classes of lipid-lowering drugs are shown in Fig. 2. The majority of patients (72.6%) received a moderate-intensity statin as first-line LLT, but only few experienced a treatment intensification. Overall, 17.4% of first LLT prescriptions were followed by a treatment modification. Among the second-line LLTs, a statin at high intensity was most frequent (42.1% of all second-line treatments). Combination therapy with statin and ezetimibe represented 19.2% of all second-line treatments. Changes in therapy to third- and fourth-line treatment were rare.

**Fig. 3:**
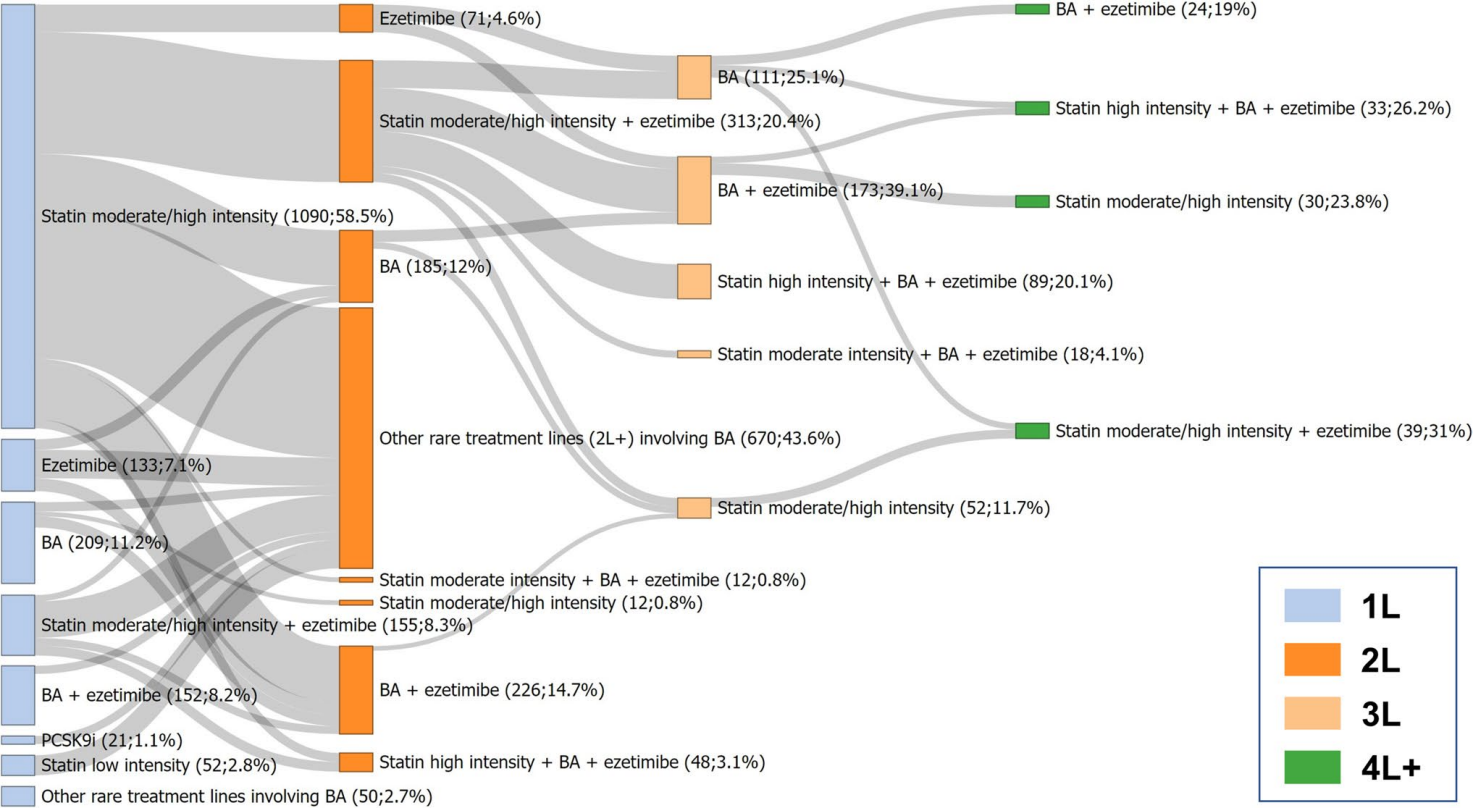
Treatment pathways of lipid-lowering therapy involving bempedoic acid. N = 1862 events of lipid-lowering therapy initiation were analysed, with double counting of patients with treatment gaps of at least one year. Rare index treatments affecting less than 1% of patients are summarized as “Other rare treatment lines involving BA”, rare 2L + treatments affecting less than 2% of patients (about n < 10 in 2L) are summarized as “Other rare treatment lines (2L +) involving BA”. *1L, 2L, …* first line, second line, …; *BA* bempedoic acid, *PCSK9i* PCSK9 inhibitor

In Fig. 3, treatment pathways of LLTs involving BA are shown. Among all treatment lines involving BA, BA was prescribed as first-line therapy in 19.4%, whereas BA monotherapy represented 11.2% and combination therapy with ezetimibe 8.2% of all first-line prescriptions. In contrast to the treatment pathways for all LLTs, for the treatment pathways involving BA, there were substantially more patients experiencing LLT modification during the treatment course. In most treatment lines involving BA, BA was prescribed as second-, third-, or fourth-line treatment, in part as monotherapy, in combination with ezetimibe, or in combination with a statin.

**Fig. 4:**
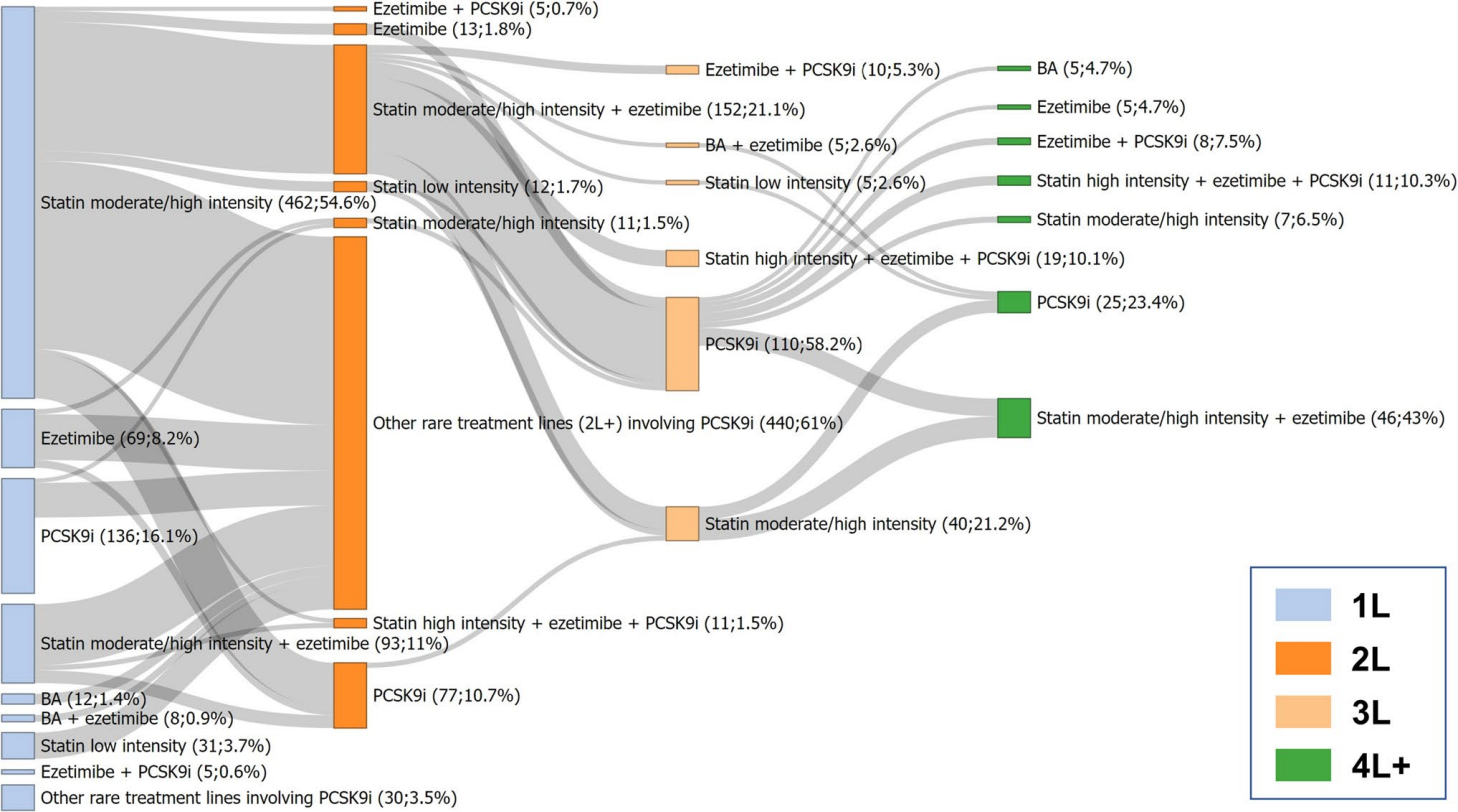
Treatment pathways of lipid-lowering therapy involving PCSK9 inhibitors. N = 846 events of lipid-lowering therapy initiation were analysed, with double counting of patients with treatment gaps of at least one year. Rare index treatments affecting less than 1% of patients are summarized as “Other rare treatment lines involving PCSK9i”, rare 2L + treatments affecting less than 5 patients are summarized as “Other rare treatment lines (2L +) involving PCSK9i”. *1L, 2L, …* first line, second line, …; *BA* bempedoic acid, *PCSK9i* PCSK9 inhibitor

Figure 4 shows all treatment pathways of LLTs involving PCSK9i. Among all treatment lines involving PCSK9i, PCSK9i were prescribed as first-line LLT as monotherapy in 16.1%. Similar to BA, PCSK9i prescriptions were mostly observed as add-on treatment or monotherapy as second-, third-, or fourth-line treatment.

### Time to switch or terminate lipid-lowering therapy

**Fig. 5:**
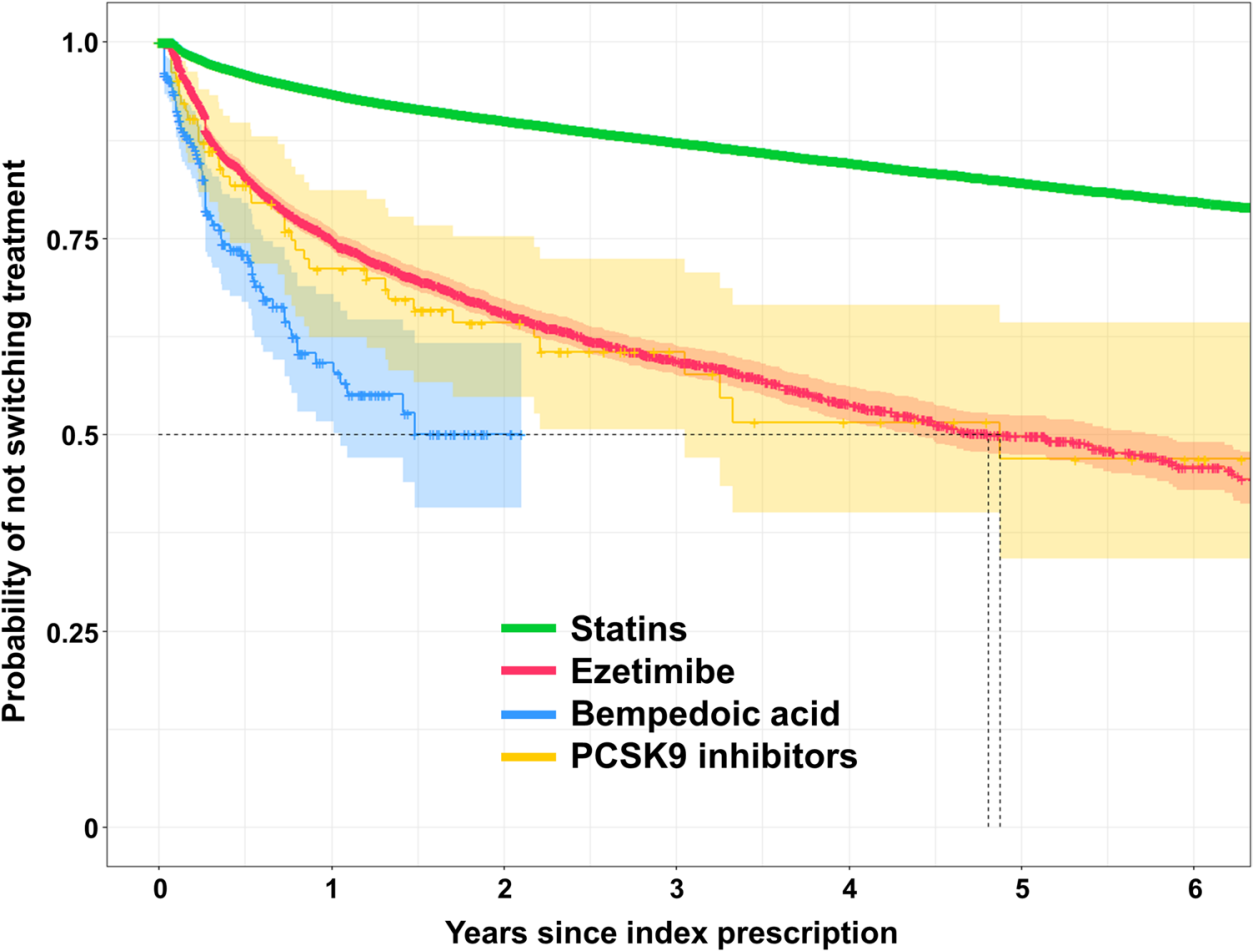
Kaplan–Meier curves of time to switch lipid-lowering therapy

The number of first prescription events for statins, ezetimibe, BA, and PCSK9i is detailed in Table 2. Among first prescriptions of statins, ezetimibe, BA, and PCSK9i, switches of therapy occurred in 4.7, 15.4, 19.0, and 23.6% of patients during the observation period, respectively (Graphical Abstract). The observation period for BA was shorter than those of the other substances due to the market launch of BA in November 2020. The median time to switch therapy was comparable for ezetimibe and PCSK9i, while the median was not reached for statins and BA. Corresponding Kaplan–Meier curves are depicted in Fig. 5.

**Table 2:** Time to treatment switch or termination for statins, ezetimibe, bempedoic acid, and PCSK9 inhibitors

	Statins	Ezetimibe	Bempedoic acid	PCSK9 inhibitors
First prescription events (n)	277,155	10,141	401	161
Treatment switch (n [%])	12,966 (4.7)	1,566 (15.4)	76 (19.0)	38 (23.6)
Time to switch treatment in years (mean [standard error])	6.02 (0.01)	4.15 (0.06)	1.01 (0.04)	3.09 (0.22)
Time to switch treatment in years (median)	–	4.81	–	4.87
Treatment termination (n [%])	135,143 (48.8)	4,418 (43.6)	136 (33.9)	56 (34.8)
Treatment continuation after break > 90 days	73,784 (26.6)	1,519 (15.0)	28 (7.0)	15 (9.3)
Treatment switch after break > 90 days	4,844 (1.7)	796 (7.8)	21 (5.2)	14 (8.7)
Treatment termination	56,515 (20.4)	2,103 (20.7)	87 (21.7)	27 (16.8)
Time to treatment termination in years (mean [standard error])	2.90 (0.01)	2.41 (0.04)	0.86 (0.04)	3.04 (0.24)
Time to treatment termination in years (median)	1.45	1.04	0.60	2.45
Kaplan–Meier analysis: no treatment termination (% [number at risk]) at 6 years	26.6 (6,845)	20.9 (131)	–	33.5 (7)

The median time to treatment switch or termination is defined as the smallest time at which the treatment persistence probability drops to 0.5. If the Kaplan–Meier curve does not drop to 0.5 or below, the median cannot be computed

**Fig. 6:**
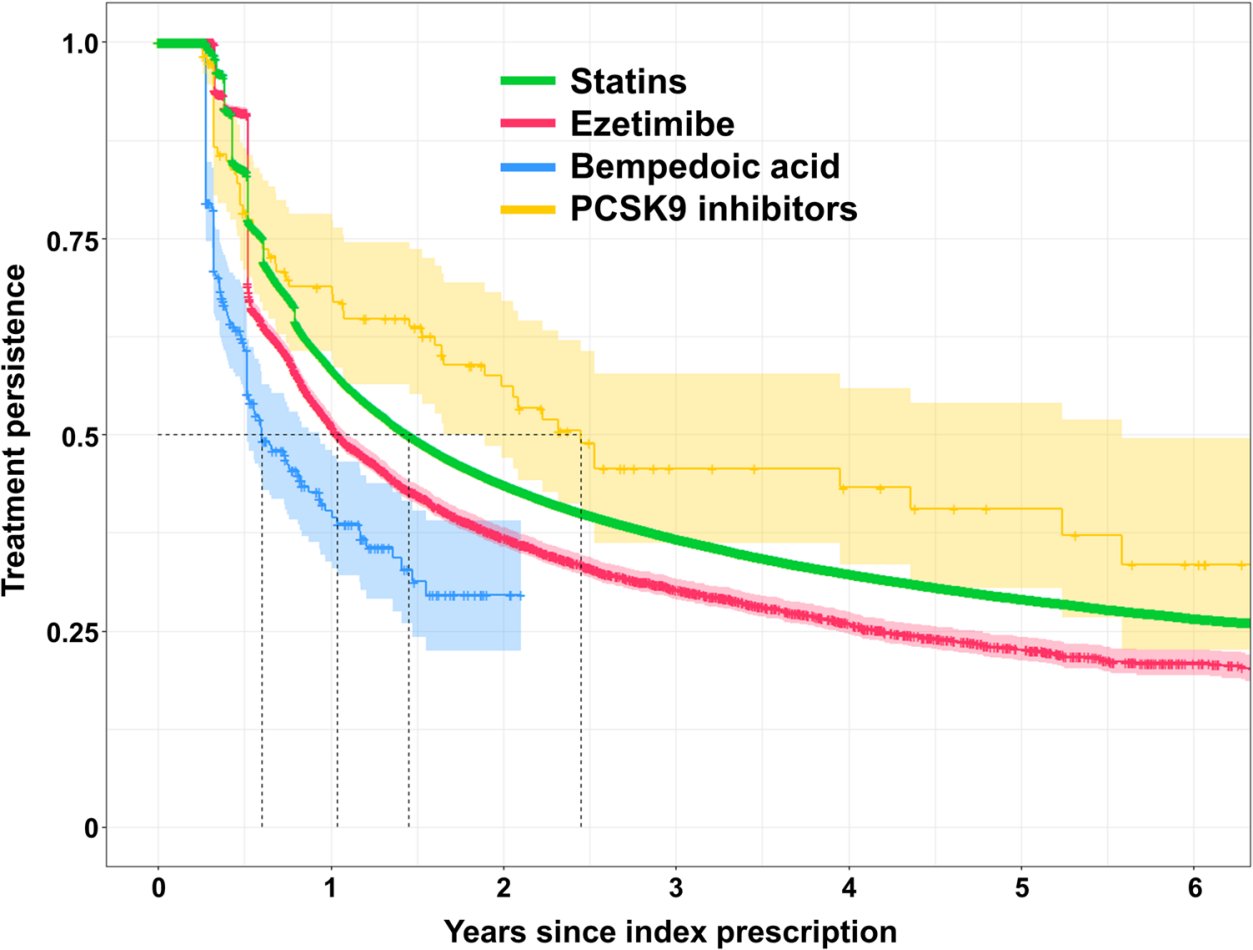
Kaplan–Meier curves of persistence to lipid-lowering therapy. Treatment persistence is defined as the probability of no treatment termination. Patients with treatment switch are excluded from this analysis

Termination of treatment with statins, ezetimibe, BA, and PCSK9i was observed in 48.8, 43.6, 33.9, and 34.8% of first prescriptions of the respective drugs. For statins, most “treatment terminations” were due to continuation of treatment after breaks of > 90 days, whereas “true” treatment termination was less frequent (26.6 vs. 20.4% of all first statin prescriptions). The opposite was the case for ezetimibe, BA, and PCSK9i (Table 2). The time to treatment termination ranged from median 0.60 years for BA to median 2.45 years for PCSK9i. The results are graphically displayed in Fig. 6.

## Discussion

This longitudinal study of 247,529 contemporary patients with high and very-high cardiovascular risk shows that three overall treatment patterns of LLTs, i.e., most patients receive moderate-intensity statins, therapy escalations occur infrequently, and medication persistence is low, have not changed compared to older reports [12–18, 20–22]. An important novel finding of this study is that BA and PCSK9i exhibit distinct patterns compared to statins and ezetimibe within the pathways of LLTs. BA and PCSK9i were prescribed only to a very small fraction of patients, mainly at later stages of the treatment course, were switched more frequently than statins and ezetimibe, but were terminated in fewer patients. Our findings suggest that there is great potential to improve LDL-C goal attainment by increasing the use of combination LLT at earlier stages and by taking steps to improve medication persistence.

In this study, among all first LLT prescriptions, 96.3% were statins as monotherapy. This is in line with previous epidemiological studies in Germany [7, 15, 20, 21] and other countries [22–24], in which approximately 90% of patients with newly initiated or established LLT had statin monotherapy. The initiation of LLT as statin monotherapy is in accordance with the 2019 ESC/EAS guidelines recommendations [1]. However, recent recommendations focus on lipid-lowering combination therapy [3]. Despite the increasing number of available LLTs and decreasing costs of medication of the generics statins and ezetimibe [21], the number of patients with escalation of their initial LLT by statin dose increase or addition of non-statin LLTs remains low, with only 4.7% of patients with statin as first LLT prescription experiencing a treatment switch in our study. These data are qualitatively similar to numbers observed e. g. half a decade ago [18].

The contemporary data show that only 26.6% of patients had not discontinued their statin after 6 years. Previous studies have reported even worse figures, for example, statin persistence of 20.6% after 3 years in Germany [15], 19% after 5 years in the United States [25], or < 25% after 2 years in Israel [12]. Of note, re-initiation of statin therapy > 90 days after runout of the previous prescription represented a substantial proportion of treatment terminations in our study, reflecting a known pattern of statin treatment [26]. Therefore, the presented proportions of patients with treatment discontinuation may underestimate supply with lipid-lowering agents in the long term.

Treatment with BA and PCSK9i mainly represented add-on treatment at later stages of the treatment course. This is in line with guideline recommendations [1]. Notably, the treatment pathways involving BA and PCKS9i therapy encompassed proportionally more treatment switches than the treatment pathways encompassing all LLTs, mainly represented by statin monotherapy. In concordance, a higher proportion of patients who received a first prescription of ezetimibe, BA, or PCSK9i experienced a treatment switch (15.4, 19.0, and 23.6%, respectively) compared to patients with first statin prescription (treatment switch in 4.7%). This may be explained by patient selection, as those initiated on ezetimibe, BA, or PCSK9i likely had received a switch of treatment before and were more likely to experience a treatment switch again. Similarly, the shorter time to treatment switch for ezetimibe, BA, and PCSK9i compared to statins may be the result of physicians and patients being inclined to switch LLT at all.

BA was switched more often after treatment initiation compared to statins and ezetimibe and, if terminated, it was terminated earlier than all other drug classes. While both BA and PCSK9i represent comparably novel options to lower LDL-C, the time to treatment termination was substantially longer for PCSK9i compared to BA. This observation may be explained by the difference in route and frequency of application. While BA is an once-daily oral drug such as statins, patients with previous side effects on statins may fear side effects of tablets with BA, too, owing to the nocebo effect of tablet intake [27]. Patient education about BA, under which muscle pain occurs in similar rates as under placebo [28], may therefore be an option to increase adherence and persistence to BA.

PCSK9i are administered subcutaneously and only every 2–4 weeks or even twice yearly, and patients may not transfer previous adverse experiences with oral drugs to PCSK9i therapy. The fact that PCSK9i are very costly, are usually not on stock in pharmacies and have specifically to be ordered in contrast to “routine” drugs may also contribute to patients expecting fewer side effects of these “special” drugs. Furthermore, in Germany, PCSK9i therapy must be initiated and supervised by specialists. Regular consultations may theoretically contribute to improved adherence and persistence to PCSK9i, whereas for oral BA as general practitioner prescription, less restrictions in terms of treatment initiation apply, and no specialized supervision is mandated. Finally, BA therapy may have preceded PCSK9i therapy in patients not at target, explaining the comparably short time to switch treatment.

Regarding PCSK9i therapy persistence, termination of treatment with PCSK9i occurred in a lower proportion of patients and later than for any other LLTs. Data of contemporary registries demonstrate persistence rates of approximately 80 to 90% over 2–3 years of PCSK9i treatment [17, 29–32], and health claims data demonstrated persistence rates of 62% after 1 year (United States) [33] and of 50.9% after 3 years [15] (Germany), compared to 68.8% and 45.7% without PCSK9i termination after 1 and 3 years, respectively, in our study.

While the population in the database is fairly representative of the total statutory health-insured population, it has to be acknowledged that privately insured patients are not covered in this analysis and might exhibit different treatment pathways. Furthermore, regional differences in atherosclerotic cardiovascular disease prevalence, prescription behaviour of physicians in different areas, and the proportions of rural vs. urban areas might also influence prescription rates and treatment pathways of lipid-lowering agents. Analyses from other German regions not covered by the database used in this study might therefore yield differing results. As example, in a previous study, the proportion of patients with a fixed-dose combination prescription of statin and ezetimibe among all statin prescriptions ranged between 1.7 and 7.0% in the 16 German federal states [21]. However, the reported proportions of prescriptions of statins and non-statins in the current study are in line with previous reports based on other databases in Germany, reinforcing the notion that our findings are generalizable at least to some extent [7, 15, 18, 20, 21, 34].

This study has several strengths. We were able to identify a large number of eligible patients from a representative and recent sample and with long follow-up. The analysis of health claims data also has limitations that have to be acknowledged. The analysed data were collected for reimbursement processing. The available data on prescriptions may not 100% concur with the medications actually taken (i.e., splitting tablets, not taking tablets), and diagnoses have not been adjudicated. The definition of treatment switches encompassed addition of a new substance or replacement by another, however it was not mandatory that treatment switches meant intensification of LLT; therefore, the proportion of treatment switches for each substance has to be interpreted in that context. Furthermore, treatment termination encompassed treatment continuation or switches after breaks of more than 90 days; some patients might have had treatment over long periods around a 90-days break and would still have been classified as “treatment termination”. As inherent limitation of claims data analyses, we were not able to identify the reasons for switching or discontinuing treatment, which could have provided valuable insights for the improvement of care. Due to the lack of laboratory results, the proportion of patients not at LDL-C target who would have required treatment intensification could not be quantified. Finally, the number of first BA and PCSK9i prescriptions was comparably low (n = 401 and n = 161 first prescription events, respectively), potentially limiting generalizability.

In summary, we found that most patients at elevated cardiovascular risk receive statin monotherapy and treatment intensification occurs infrequently. BA and PCSK9i were prescribed mainly at later stages of the treatment course, and were switched more frequently than statins or ezetimibe. The time to treatment termination was longest for PCSK9i, and shortest for BA. There is great potential to improve LDL-C target attainment in patients at elevated cardiovascular risk by better utilizing combination LLTs and supporting patients to adhere to their treatment in particular in the long term.

## Supplementary Information

Below is the link to the electronic supplementary material.

Supplementary file1 (PDF 160 KB)

## Data Availability

The data underlying this article were provided by Gesundheitsforen Leipzig GmbH. The data can be obtained from Gesundheitsforen Leipzig GmbH on request (https://www.gesundheitsforen.net/).
