# Supplementary material for: Treatment pathways of lipid-lowering therapies in Germany 2016–2022
Source: Clin Res Cardiol. 2025 May 28;115(2):266–76. doi: 10.1007/s00392-025-02686-5 (PMC12823755; doi:10.1007/s00392-025-02686-5)
Supplement: Supplementary file 1 — Supplementary file1 (PDF 160 KB) [file 392_2025_2686_MOESM1_ESM.pdf]

Supplementary material

Table of contents

Table S1: ATC codes of lipid-lowering agents 2

Table S2: Definition of statin intensity for average daily doses 3

Table S3: ICD-10/OPS codes used for the definition of high or very-high cardiovascular risk 4

Supplement to:

Treatment pathways of lipid-lowering therapies in Germany 2016–2022

Julius L. Katzmann, Claudia Grellmann, Beate Leppert, Irina Müller-Kozarez, Martin Schulz, Ulrich Laufs

Table S1: ATC codes of lipid-lowering agents

ATC code	Active substance
**C10AX15**	Bempedoic acid
**C10BA10**	Bempedoic acid and ezetimibe
**C10AX09**	Ezetimibe
**C10BA03**	Pravastatin and fenofibrate
**C10AX13**	Evolocumab
**C10AX14**	Alirocumab
**C10AX16**	Inclisiran
**C10AA01**	Simvastatin
**C10AA02**	Lovastatin
**C10AA03**	Pravastatin
**C10AA04**	Fluvastatin
**C10AA05**	Atorvastatin
**C10AA07**	Rosuvastatin
**C10AA08**	Pitavastatin
**C10BX03**	Atorvastatin and amlodipine
**C10BX05**	Rosuvastatin and acetylsalicylic acid
**C10BX06**	Atorvastatin, acetylsalicylic acid and ramipril
**C10BX09**	Rosuvastatin and amlodipine
**C10BX11**	Atorvastatin, amlodipine and perindopril
**C10BX18**	Atorvastatin, amlodipine and ramipril
**C10BA02**	Simvastatin and ezetimibe
**C10BA05**	Atorvastatin and ezetimibe
**C10BA06**	Rosuvastatin and ezetimibe

Table S2: Definition of statin intensity for average daily doses

Statin	Low intensity (LDL-C reduction < 30%)	Moderate intensity (LDL-C reduction ≥ 30% to < 50%)	High intensity (LDL-C reduction ≥ 50%)
**Atorvastatin**	–	< 30 mg	≥ 30 mg
**Fluvastatin**	< 60 mg	≥ 60 mg	–
**Lovastatin**	< 30 mg	≥ 30 mg	–
**Pitavastatin**	< 1.5 mg	≥ 1.5 mg	–
**Pravastatin**	< 30 mg	≥ 30 mg	–
**Rosuvastatin**	–	< 15 mg	≥ 15 mg
**Simvastatin**	< 15 mg	15 mg–59 mg	≥ 60 mg

Classification according to Fox et al [18]. LDL-C: Low-density lipoprotein cholesterol.

Table S3: ICD-10/OPS codes used for the definition of high or very-high cardiovascular risk

ICD-10 codes

Code	Description
**E10.2**	Diabetes mellitus type 1 with renal complications
**E10.3**	Diabetes mellitus type 1 with ophthalmic complications
**E10.4**	Diabetes mellitus type 1 with neurological complications
**E10.5**	Diabetes mellitus type 1 with peripheral vascular complications
**E10.7**	Diabetes mellitus type 1 with multiple complications
**E11.2**	Diabetes mellitus type 2 with renal complications
**E11.3**	Diabetes mellitus type 2 with ophthalmic complications
**E11.4**	Diabetes mellitus type 2 with neurological complications
**E11.5**	Diabetes mellitus type 2 with peripheral vascular complications
**E11.7**	Diabetes mellitus type 2 with multiple complications
**E12.2**	Malnutrition-related diabetes mellitus with renal complications
**E12.3**	Malnutrition-related diabetes mellitus with ophthalmic complications
**E12.4**	Malnutrition-related diabetes mellitus with neurological complications
**E12.5**	Malnutrition-related diabetes mellitus with peripheral vascular complications
**E12.7**	Malnutrition-related diabetes mellitus with multiple complications
**E13.2**	Other specified diabetes mellitus with renal complications
**E13.3**	Other specified diabetes mellitus with ophthalmic complications
**E13.4**	Other specified diabetes mellitus with neurological complications
**E13.5**	Other specified diabetes mellitus with peripheral vascular complications
**E13.7**	Other specified diabetes mellitus with multiple complications
**E14.2**	Unspecified diabetes mellitus with renal complications
**E14.3**	Unspecified diabetes mellitus with ophthalmic complications
**E14.4**	Unspecified diabetes mellitus with neurological complications
**E14.5**	Unspecified diabetes mellitus with peripheral vascular complications
**E14.7**	Unspecified diabetes mellitus with multiple complications
**G45**	Cerebral transient ischemia and related syndroms
**I10**	Essential (primary) hypertension
**I11**	Hypertensive heart disease
**I12**	Hypertensive renal disease
**I13**	Hypertensive heart and renal disease
**I15**	Secondary hypertension
**I20**	Angina prectoris

**I21**	Acute myocardial infarction
**I22**	Recurrent myocardial infarction
**I23**	Acute complications after acute myocardial infarction
**I24**	Other acute ischaemic heart disease
**I25**	Chronic ischaemic heart disease
**I63**	Cerebral infarction
**I64**	Stroke, not specified as haemorrhage or infarction
**I65**	Occlusion and stenosis of pre-cerebral arteries without resulting stroke
**I66**	Occlusion and stenosis of cerebral arteries without resulting stroke
**I70**	Atherosclerosis
**I73.9**	Peripheral vascular disease
**I74**	Arterial embolism and thrombosis
**N18.3***	Chronic kidney disease, stage 3
**N18.4**	Chronic kidney disease, stage 4
**N18.5**	Chronic kidney disease, stage 5
**Z95.1**	Presence of aortocoronary bypass graft
**Z95.5**	Presence of coronary angioplasty implant and graft
**(E10.0 or E10.1 or E10.6 or E10.8 or E10.9) and (F17 or Z72.0)***	Diabetes mellitus type 1 without organ damage in combination with smoking
**(E11.0 or E11.1 or E11.6 or E11.8 or E11.9) and (F17 or Z72.0)***	Diabetes mellitus type 2 without organ damage in combination with smoking
**(E12.0 or E12.1 or E12.6 or E12.8 or E12.9) and (F17 or Z72.0)***	Malnutrition-related diabetes mellitus without organ damage in combination with smoking
**(E13.0 or E13.1 or E13.6 or E13.8 or E13.9) and (F17 or Z72.0)***	Other specified diabetes mellitus without organ damage in combination with smoking
**(E14.0 or E14.1 or E14.6 or E14.8 or E14.9) and (F17 or Z72.0)***	Unspecified diabetes mellitus without organ damage in combination with smoking

OPS codes

Procedure	Codes
**Coronary artery bypass grafting**	5-360, 5-361, 5-362, 5-363, 5-364, 5-369
**Percutaneous coronary intervention**	8-837.0, 8-837.1, 8-837.2, 8-837.5, 8-837.k, 8-837.m, 8-837.w, 8-837.x, 8-837.y
**Peripheral arterial disease-related procedures**	8-836.0a, 8-836.0c, 8-836.0h, 8-836.0j, 8-836.0k, 8-836.0m, 8-836.0n, 8-836.0p, 8-836.0q, 8-836.0r, 8-836.0s, 8-836.3c, 8-836.3k, 8-840.0a, 8-840.0c, 8-840.0h, 8-840.0j, 8-840.0k, 8-840.0m, 8-840.0n, 8-840.0p, 8-840.0q, 8-840.0r, 8-840.0s, 8-840.1a, 8-840.1c, 8-840.1h, 8-840.1j, 8-840.1k, 8-840.1m, 8-840.1n, 8-840.1p, 8-840.1q, 8-840.1r, 8-840.1s, 8-840.2a, 8-840.2c, 8-840.2h, 8-840.2j, 8-840.2k, 8-840.2m, 8-840.2n, 8-840.2p, 8-840.2q, 8-840.2r, 8-840.2s, 8-840.3a, 8-840.3c, 8-840.3h, 8-840.3j, 8-840.3k, 8-840.3m, 8-840.3n, 8-840.3p, 8-840.3q, 8-840.3r, 8-840.3s, 8-840.4a, 8-840.4c, 8-840.4h, 8-840.4j, 8-840.4k, 8-840.4m, 8-840.4n, 8-840.4p, 8-840.4q, 8-840.4r, 8-840.4s, 8-840.5a, 8-840.5c, 8-840.5h, 8-840.5j, 8-840.5k, 8-840.5m, 8-840.5n, 8-840.5p, 8-840.5q, 8-840.5r, 8-840.5s, 8-841.0a, 8-841.0c, 8-841.0h, 8-841.0j, 8-841.0k, 8-841.0m, 8-841.0n, 8-841.0p, 8-841.0q, 8-841.0r, 8-841.0s, 8-841.1a, 8-841.1c, 8-841.1h, 8-841.1j, 8-841.1k, 8-841.1m, 8-841.1n, 8-841.1p, 8-841.1q, 8-841.1r, 8-841.1s, 8-841.2a, 8-841.2c, 8-841.2h, 8-841.2j, 8-841.2k, 8-841.2m, 8-841.2n, 8-841.2p, 8-841.2q, 8-841.2r, 8-841.2s, 8-841.3a, 8-841.3c, 8-841.3h, 8-841.3j, 8-841.3k, 8-841.3m, 8-841.3n, 8-841.3p, 8-841.3q, 8-841.3r, 8-841.3s, 8-841.4a, 8-841.4c, 8-841.4h, 8-841.4j, 8-841.4k, 8-841.4m, 8-841.4n, 8-841.4p, 8-841.4q, 8-841.4r, 8-841.4s, 8-841.5a, 8-841.5c, 8-841.5h, 8-841.5j, 8-841.5k, 8-841.5m, 8-841.5n, 8-841.5p, 8-841.5q, 8-841.5r, 8-841.5s, 8-843.0a, 8-843.0c, 8-843.0h, 8-843.0j, 8-843.0k, 8-843.0m, 8-843.0n, 8-843.0p, 8-843.0q, 8-843.0r, 8-843.0s, 8-843.1a, 8-843.1c, 8-843.1h, 8-843.1j, 8-843.1k, 8-843.1m, 8-843.1n, 8-843.1p, 8-843.1q, 8-843.1r, 8-843.1s, 8-843.2a, 8-843.2c, 8-843.2h, 8-843.2j, 8-843.2k, 8-843.2m, 8-843.2n, 8-843.2p, 8-843.2q, 8-843.2r, 8-843.2s, 8-843.3a, 8-843.3c, 8-843.3h, 8-843.3j, 8-843.3k, 8-843.3m, 8-843.3n, 8-843.3p, 8-843.3q, 8-843.3r, 8-843.3s, 8-843.4a, 8-843.4c, 8-843.4h, 8-843.4j, 8-843.4k, 8-843.4m, 8-843.4n, 8-843.4p, 8-843.4q, 8-843.4r, 8-843.4s, 8-843.5a, 8-843.5c, 8-843.5h, 8-843.5j, 8-843.5k, 8-843.5m, 8-843.5n, 8-843.5p, 8-843.5q, 8-843.5r, 8-843.5s, 8-844.0c, 8-844.1c, 8-844.2c, 8-844.3c, 8-844.4c, 8-844.5c

Notes: * used to define high cardiovascular risk, all others used to define very-high cardiovascular risk according to the 2019 ESC/EAS guidelines for the management of dyslipidaemias [1].
